# Mixed-method examination of factors associated with adolescent decision-making and involvement in care in the context of advanced cancer

**DOI:** 10.1017/S1478951524000026

**Published:** 2024-02-14

**Authors:** Malcolm Sutherland-Foggio, Anna L. Olsavsky, Micah A. Skeens, Leena Nahata, Kylie Hill, Megan Schaefer, Alexandra Himelhoch, Ansley E. Kenney, Lisa Humphrey, Randal Olshefski, Cynthia A. Gerhardt

**Affiliations:** 1 Center for Biobehavioral Health, The Research Institute at Nationwide Children’s Hospital, Columbus, OH, USA; 2 Nationwide Children’s Hospital, Columbus, OH, USA; 3 Department of Pediatrics, The Ohio State University College of Medicine, Columbus, OH, USA; 4 University of Florida Department of Psychology, Gainsville, FL, USA; 5 Department of Psychology, University of Wisconsin-Milwaukee, Milwaukee, WI, USA

**Keywords:** Adolescent, chronic disease, neoplasms, decision-making, mixed-method

## Abstract

**Objectives.:**

Adolescents with cancer often experience significant symptom burden and aggressive treatment near end-of-life. Increased adolescent involvement in care and decision-making may benefit health outcomes. Limited research has examined factors associated with adolescents’ involvement in care in the context of advanced disease. Thus, we examined the impact of background factors and decision-making perceptions on both adolescents’ involvement in care and their desired change in involvement.

**Methods.:**

Adolescents with advanced cancer (<60% survival or refractory/relapsed disease), ages 10–23 (*n*= 41; *M*_*age*_ = 15.37), were recruited approximately 1 month after diagnosis to complete measures of decision-making perceptions and their family role. Hierarchical regressions examined the contributions of background factors and decision-making perceptions to adolescents’ frequency and desired involvement in their care. Qualitative interviews regarding decision-making were analyzed using deductive analysis.

**Results.:**

The model examining frequency of involvement in care was significant, *F*(5,34) = 3.12, *p* = .02, *R*^*2*^ = .31. Older age was the only significant predictor (*β* = .13, *p* = .003). The model examining desired involvement was non-significant, *F*(5,34) = 2.22, *p* = .075. Qualitative analysis indicated that (1) older adolescents have more involvement in decision-making, (2) collaborative decision-making occurred between the adolescent and extended family, and (3) adolescents trusted others to make decisions. Integration of qualitative and quantitative data revealed congruence in findings.

**Significance of results.:**

Adolescents with advanced cancer, who consider how decisions directly impact them and prefer greater autonomy, may be more involved in their medical care. Research is needed to identify other longitudinal predictors of decision-making and involvement in care. Providers should consider encouraging families to communicate their preferences and engage in shared decision-making.

Survival rates for pediatric cancers have increased to over 85% (Siegel et al. 2022). However, survival rates for some malignancies are still below 50%, and cancer remains the leading cause of disease-related death in children (Siegel et al. 2022). For youth with advanced cancer, quality of life (QOL) can decline due to increased symptom burden and stress, particularly as the child approaches end-of-life (EOL) (Kaasa and Loge 2003; Montgomery et al. 2020). Further, families of children with advanced cancer may face many difficult decisions that can affect the child’s QOL (Foster et al. 2010). For adolescents, these decisions come at a time when, developmentally, they desire increased autonomy but may become increasingly dependent on their parents due to illness (Daddis 2011; Shifflet-Chila et al. 2016).

Medical decisions can include aggressive treatments to extend life, experimental therapies, palliative care, and/or hospice/EOL care (Epelman 2012; Foster et al. 2010). Each of these choices presents its own challenges, particularly regarding adolescent involvement in shared decision-making. Treatment decisions are generally made by parents, who may defer to their child’s physician (Sisk et al. 2019), potentially due to limited knowledge (Day et al. 2016). Further, some parents may exhibit protective and controlling behavior, further limiting adolescents’ decisional autonomy (Davies et al. 2015). Importantly, however, when adolescents with cancer are more involved in treatment decisions, they may have improved health outcomes, specifically better treatment adherence and, subsequently, higher health-related QOL (Shoshani and Kanat-Maymon 2018). To date, little is known about factors related to adolescents’ involvement in their care and decision-making in the context of advanced cancer.

Qualitative and quantitative research has identified situational (e.g., decision seriousness and symptom burden), informational (e.g., understanding of the illness), and parent and provider (e.g., relationships, roles, and perspectives) factors as contributors to adolescent desire for involvement in treatment decisions (Coyne et al. 2014; Knopf et al. 2008; Miller 2009; Pyke-Grimm et al. 2019; Snaman et al. 2021a). Older age may be associated with higher engagement and agreement with parents in decision-making (Miller 2009); however, adolescents in the later stages of development may have more disagreements or conflict with their parents (Pyke-Grimm et al. 2019). Given this contradiction, Pyke-Grimm et al. (2019) suggest time since diagnosis as an alternative predictor of involvement.

Limited work has investigated the specific factors adolescents with chronic illness consider during their decision-making process and how it may contribute to their level of involvement (Lipstein et al. 2014; Snaman et al. 2021a). Lipstein et al. (2014) found that adolescents with chronic illness may focus on factors related to short-term QOL, while parents focus on long-term outcomes. Additionally, parental withholding of information may preclude adolescents’ thorough assessment of factors and choices (Davies et al. 2015; Lipstein et al. 2014). Adolescents and young adults with cancer prefer to be active participants in treatment decision-making, but there is notable variation in the factors considered most important, including time until cancer progression, QOL, and side effects (Snaman et al. 2021a).

Considering gaps in the literature and potential for greater adolescent involvement in decision-making to improve treatment adherence and QOL, this mixed-method, pilot study examined factors related to adolescents’ involvement in care and their desire for change in their involvement in the context of advanced cancer. Our study includes 3 primary aims: (1) describe adolescent perceptions of decision-making, frequency of and desire for involvement in their care both quantitatively and qualitatively, (2) quantitatively examine the role of demographic characteristics, medical factors, and decision-making perceptions in adolescents’ frequency of and desire for involvement in their care, and (3) integrate quantitative and qualitative data to provide a more comprehensive representation of adolescents’ roles in their medical care.

## Methods

This work was conducted as part of a larger study examining goals and decision-making among children with advanced cancer and their caregivers. The overarching study included surveys and interviews with families at enrollment, 6 months, and 12 months, in addition to monthly online symptom surveys. The following data were collected at enrollment visits between August 2017 and April 2022.

### Participants

Families were eligible to participate in the larger study if the child (1) had advanced cancer (either relapsed/refractory disease or prognosis <60% as estimated by their physician), (2) was 5–25 years old, (3) had at least 1 English-speaking caregiver, and (4) lived <150 miles from the hospital. Children with significant developmental disabilities were excluded from the study.

### Sample characteristics

Adolescents (*N* = 41) were aged 10–23 years old (*M* = 15.37, *SD* = 3.34). Recognizing that eligibility criteria included children across developmental periods, we use the term adolescents as existing literature suggests adolescence to include ages 10–24 years old (Sawyer et al. 2018). The sample was mostly male (65.9%) and White (80.5%). The most common diagnoses were “other solid tumors” (48.8%) (see Table 1 for additional demographics).

### Procedures

Following approval from the Institutional Review Board (IRB16–00869), research staff identified eligible families at a large United States Midwestern children’s hospital through screening medical charts and palliative care and oncology consults. Eligible families were contacted via phone or in-person approximately 1 month following their advanced cancer diagnosis to introduce the study and, if interested, written informed consent/assent was obtained. Staff then scheduled a time to complete enrollment at the hospital, at home, or virtually (for assessments during the COVID-19 pandemic). Each adolescent and parent received $40 in compensation for the initial enrollment visit.

### Measures

#### Demographic questionnaire

A demographic form collected adult-report (age >18 years) of family background information, such as age, date of birth, race, ethnicity, education level, income, and religious affiliation.

#### Cancer Information Questionnaire for Adolescents (C-CIQ)

This questionnaire was previously developed by members of the study team to assess adolescent knowledge of their prognosis, goals of care, and the factors considered when making decisions about care (Shultz et al. 2017). Adolescents >10 provided self-perceptions of 5-year disease-free survival on a 0–100% scale, where they received this information (e.g., oncologist, parents, and internet), and their perceptions of their parent’s and oncologist’s prognosis estimate. Goals of care were assessed through an open-ended written question (i.e., “What are your current medical or treatment goals for your care?”), as well as questions rated from 1 to 5 (“Strongly Disagree” to “Strongly Agree”) regarding how much the adolescent agrees with their parents and health-care team on the goals of care.

#### Decision-making survey

Developed by members of the study team, factors considered in decision-making were assessed using a 17-item Likert-type questionnaire including intrinsic factors (e.g., “Symptoms [e.g. pain, nausea],” “My current/future quality of life,” and “How much time I would have to be at the hospital”) and extrinsic factors (e.g., “Being a good son/daughter,” “The financial impact on my family,” “What my parents or family thinks,” and “What my health-care team thinks”). Items were rated from 0 to 4 (“not at all” to “a lot”). The internal consistency for intrinsic (*α* = .79) and extrinsic (*α* = .80) subscales was acceptable for this sample.

#### Autonomy and Information-Seeking Preference Scale (AISPS)

The AISPS assessed adolescent preferences for autonomy in their medical care and the degree to which they desired information about their medical care and condition (Ende et al. 1989; Simon et al. 2010). Two subscales measured attitudes towards autonomous decision-making and information-seeking. Internal consistency and validity for this measure have been established (Ende et al. 1989). Internal consistency for the AISPS (*α* = .75), as well as the decision-making (*α* = .68) and information-seeking (*α* = .64) subscales, was acceptable for this sample.

#### Family Roles Questionnaire (FRQ)

This measure was adapted from previous work (Quittner et al. 1992) and assessed the frequency of parent and child involvement in medical care, as well as their current satisfaction with their role and whether they would like to be involved more or less. The FRQ measured involvement in 3 domains: “handling your day-to-day medical care,” “talking with the medical team,” and “making decisions about your treatment.” Frequency for each of these items is rated from 0 to 4 (“My parents usually do all of it” to “I usually do all of it”). Role satisfaction is rated from 0 to 3 (“Not at all” to “Very”). Desired change in involvement is rated from 0 to 2 scale with options of desires for “Less,” “The same,” or “More” involvement. Internal consistency for the frequency (*α* = .70), satisfaction (*α* = .89), and involvement (*α* = .71) subscales was acceptable for this sample.

### Qualitative interview

#### Family decision-making

Caregivers and adolescents were interviewed separately using a structured 8-question open-ended interview regarding goals and decision-making. Interview questions were developed by study investigators and tested with 10 families of children with cancer. Research staff at the postdoctoral, masters, and postbaccalaureate level conducted interviews under the training and supervision of a doctoral-level psychologist. This manuscript examined responses to: “How has your family made decisions about your care? For example, who is involved in making treatment decisions, and what factors are considered when making these choices?” All interviews were digitally recorded and transcribed for coding.

### Analyses

#### Quantitative analyses

Descriptive statistics were used to characterize adolescent background characteristics, medical factors, autonomy preferences, involvement in care, decision-factors, and agreement with parents on goals of care. Point-biserial and Pearson correlations examined associations between variables of interest with both involvement in care and desire for change in involvement. Significant correlates were then added to 2 hierarchical linear regression models which examined the following predictors of decision involvement (model 1) and desired change in involvement (model 2): (1) adolescent age, (2) autonomy attitudes, (3) decision-making factors, and (4) agreement with parents on goals of care. For model 1, post hoc power analyses revealed over 80% power to detect identified effect sizes. However, for model 2, post hoc power analyses revealed that we were underpowered (67%) to detect observed effect sizes.

#### Qualitative analyses

Qualitative data analysis followed deductive coding techniques. Decision-making process codes were derived from previous literature in adolescents with cancer and their families (Darabos et al. 2021; Kelly et al. 2017; Miller 2018; Pyke-Grimm et al. 2006). Literature revealed a continuum of 3 types of adolescent involvement in decision-making: active decision involvement, collaborative decision involvement, and passive decision involvement. Active decision involvement and collaborative decision involvement both fall on the continuum of shared decision-making (Kon 2010). Decision-making factor codes were derived from items included in the C-CIQ. Similar factors were combined into single codes for efficiency, and the codes were split into groups of intrinsic and extrinsic factors as they were for the C-CIQ. Decision-making process codes and decision-making factor codes are further defined in Table 2. Interview responses were coded independently in 2 groups based on the median age of participants: <16 (*n* = 14) or ≥16 (*n* = 16).

Four researchers (MSS, ALO, LN, and MAS), experienced in qualitative coding, reviewed participants’ responses and independently discerned the presence or absence of each code based on their interpretation of the adolescent’s response. Disagreements in coding were settled through re-reading and deliberation to reach consensus. Following this iterative process, coders discussed the themes presented by the data. Based on these discussions, the primary author derived themes for each group of codes.

## Results

### Quantitative results

Adolescents with advanced cancer did not desire a change in their level of involvement (scale: 0–2; *M* = 1.05; *SD* = .39), were highly satisfied with their decision-making role (scale: 0–3; *M* = 2.72; *SD* = .59), and were not frequently involved in their care (scale: 0–4; *M* = 1.70; *SD* = .77). However, correlations revealed that older adolescents were more frequently involved in their care, *r*(41) = .50; *p* < .001. No association was found between care involvement and time since diagnosis.

Regarding decision-making factors, adolescents tended to value intrinsic factors (*M* = 3.29; *SD* = .84) more than extrinsic factors (*M* = 2.62; *SD* = 1.13), *t*(40) = 3.28, *p* = .002. Greater consideration of intrinsic factors was associated with older adolescent age, *r*(41) = .47, *p* = .002, and a stronger preference for autonomy in medical decisions, *r*(41) = .39, *p* = .012. Consideration of extrinsic factors was not associated with any background or decision-making variables. Adolescents also reported a high level of agreement with their parents regarding goals of care (*M* = 4.80; *SD* = .41).

On average, adolescents did not have strong opinions regarding the level of autonomy they should have when making medical decisions (scale: 1–5; *M* = 3.12; *SD* = .73). However, adolescents strongly believed that they should be informed regarding their medical status (*M* = 4.34; *SD* = .42). Older adolescents tended to believe that they should have more autonomy when making medical decisions, *r*(41) = .39, *p* = .002, and they should be well informed regarding their condition, *r*(41) = .31, *p* = .046. Female adolescents held stronger beliefs that they should have autonomy when making medical decisions, *t*(39) = −2.12, *p* = .04, *d* = −.68.

Details of the steps for each hierarchical linear regression model can be found in Table 3. Adolescent age was used as a predictor instead of time since initial diagnosis, because the latter was not significantly associated with any other variables included in analyses. The overall model examining frequency of adolescent involvement in decision-making was significant, *F*(5,34) = 3.12, *p* = .02, and predicted 31.4% of the variance in the frequency of involvement. Older age (*β* = .13, *p* = .003) was the only significant predictor of greater frequency of adolescent involvement in the final model. The model examining adolescent desire for change in decision-making involvement was significant at the third step; however, the addition of agreement with parents on the goals of care in the final step resulted in non-significance, *F*(5,34) = 2.22, *p*= .075, explaining 24.6% of the variance in desired change in decision-making involvement. Significant predictors in the final model included intrinsic factors (*β* = −.44, *p* = .02) and, marginally, the adolescent’s preference for autonomous decision-making (*β* = .37, *p* = .055) after controlling for adolescent age (*β* = .26, *p* = .15).

### Qualitative results

Qualitative analysis of the question “How has your family made decisions about your care? For example, who is involved in making treatment decisions, and what factors are considered when making these choices?” revealed 3 themes, which are presented in order from most to least involvement in decision-making: (1) older adolescents had a higher level of involvement, (2) collaborative decision-making occurred between adolescents, across age groups, and their extended family, and (3) adolescents, across age groups, trusted others to make decisions. Only about half of the interviews (*n* = 15) included data on decision factors. These participants emphasized potential side effects and the effectiveness of treatment as the most important factors considered in decision-making.

#### Older adolescents had a high level of involvement

Five participants, all of whom were over age 16, described themselves as highly active in decision-making. They often described how their parents have some involvement in treatment decisions, but they primarily made final decisions. A small number (*n* = 2) of older adolescents stated that their parents had little to no involvement in their treatment decision-making.

#### Collaborative decision-making occurred between the adolescents, across age groups, and their extended family

Over half of the adolescents (*n* = 16) described their decision-making process as a collaboration between themselves, their parents, and their extended family. Adolescents who reported this theme were split evenly between the above 16 and under 16 coding groups and primarily referenced collaborating with their primary caregivers to make decisions. Others discussed how extended family may be involved, particularly if they have experience in medicine or caring for someone with cancer.

#### Adolescents, across age groups, trusted others to make decisions

Some adolescents (*n* = 9) described themselves as having a passive role in their treatment decision-making, trusting their loved ones to make medical decisions on their behalf. All but one of the participants who endorsed this theme were under 16 years old. Some preferred less involvement in decision-making, while others recognized their family as having more understanding and wisdom. Some participants (*n* = 2) noted that they and their family placed their trust entirely in their health-care team’s recommendations.

### Mixed-method results

Integration of quantitative and qualitative data revealed congruency in adolescents’ level of involvement in their care. Older adolescents were more likely to qualitatively describe themselves as highly involved in making decisions about their care, *F*(2,27) = 10.36, *p* < .001. Adolescents who placed higher importance on intrinsic factors tended to qualitatively report themselves as being collaboratively or actively involved in decisions regarding their medical care, *F*(1,28) = 5.41, *p* = .03. Their qualitative self-report of decision involvement also aligned with quantitative self-report of the frequency of involvement in treatment decisions, *F*(2,27) = 5.64, *p* = .009. Differences by group can be seen in Table 4.

## Discussion

This mixed-method pilot study provides novel insights into adolescents’ involvement in their medical care and decision-making in the context of advanced cancer. Findings give voice to these adolescents, who are typically underrepresented in research, and provide insight into the understudied area of adolescent decision-making in medical care. We identified several factors related to adolescents’ involvement and desire for change in care and decision-making involvement. Adolescents were, on average, not deeply involved in their care and were satisfied with this level of involvement. However, older adolescents were more involved, sought more autonomy in decision-making, and placed greater weight on intrinsic factors that included how decisions directly affected them. Concordantly, relative to younger adolescents, older adolescents described themselves as more involved in decision-making and also placed more importance on intrinsic factors in decision-making, as depicted in their qualitative reports.

Older age appeared to be particularly relevant when examining frequency of involvement, as it was the only significant predictor in the final regression model, which explained almost a third of the variance. This finding furthers the case made by previous work (Snaman et al. 2021a) regarding the importance of developmental status when seeking to understand adolescent involvement in their cancer care. For example, parents may withhold information to protect their adolescent, or providers may only address parents regarding disease information and decision-making regardless of patient age (Davies et al. 2015). Changing parental and provider behavior may serve as impactful points of intervention to support increased adolescent participation in their care and, subsequently, improve adolescent health outcomes (Coyne et al. 2016; Shoshani and Kanat-Maymon 2018).

Integration of quantitative and qualitative results provides additional support to the importance of intrinsic decision factors and developmental stage when understanding the degree of involvement adolescents with advanced cancer have and desire. Placing value on intrinsic factors may indicate adolescents’ increased understanding regarding how treatment decisions can impact their well-being. Further research in this area can help shed light on how consideration of intrinsic and extrinsic factors in decision-making changes during development.

However, two-thirds of the variance in outcomes remained unexplained by the regression models. Given that other factors, such as disease status, treatment stage, and symptom burden, may also play a role in involvement beyond that of age (Kelly et al. 2017; Weaver et al. 2015), further research is needed to characterize the context of adolescent involvement in their medical care and decision-making. In slight contrast, our results indicated that adolescents had less desire for change in their role if they placed greater value on intrinsic factors (which include symptom and disease status factors) when controlling for age. This was particularly interesting as higher value of intrinsic factors was positively associated with greater autonomy preference, while more autonomy preference was related to an elevated desire for change in involvement. A reason for this may be that some adolescents with advanced cancer, despite seeking a role in the decision-making process, may not desire the responsibility of making important treatment-related decisions, due to the weight of the decision (Weaver et al. 2015). Thus, the authors encourage collaborative decision-making between adolescent patients, caregivers, and medical teams as a way to ameliorate adolescents’ potential distress, pressure, and responsibility associated with making treatment decisions.

These findings were also evident qualitatively; most adolescents reported using a collaborative approach to decision-making and managing their care. With over half of participants under 18 years old, this finding was not particularly surprising. However, other qualitative studies in pediatric cancer have found adolescents reported being more actively involved (Ruhe et al. 2016; Weaver et al. 2015). The discrepancy in these findings may indicate the complex dynamics in which adolescents participate in their care, specifically with respect to advanced cancer (Smith et al. 2020).

Nevertheless, this study has limitations. This work was conducted with a unique sample of adolescents with advanced cancer. Pandemic-related restrictions disrupted data collection which limited sample size. The study population was largely White and male and included a broad age range. Additionally, we were slightly underpowered for the full hierarchical regression models. Furthermore, there is a lack of well-established measures to assess decision-making in pediatric advanced cancer. Future qualitative work may provide a richer perspective from adolescents and inform the development of quantitative instruments. Research with larger, more diverse samples is needed to confirm our findings and identify other cultural factors that may influence decision-making in families. Further, longitudinal work can examine how these decision-making processes may change as adolescents mature, gain more experience with their disease, or as their disease progresses.

Our findings provide guidance on the clinical care for adolescents with advanced cancer. Inclusion of adolescents in discussions about their disease can help increase involvement in their care (Smith et al. 2020) and comfort in discussing their wishes with their family outside of the hospital context. Clinicians should sensitively assess adolescents’ desired level of involvement and the factors they consider and prioritize when making decisions about their treatment. Decision-making tools are available to improve care by involving adolescents in identifying what is important to them when managing their advanced cancer (Lyon et al. 2022; Nahata et al. 2020; Snaman et al. 2021b; Wu et al. 2021; Zadeh et al. 2015). Communication and assessment of preferences and goals of care should be an ongoing process, as these can evolve as the disease progresses. Facilitating active involvement of adolescents in their care can result in improved health outcomes (Shoshani and Kanat-Maymon 2018). In summary, our work highlights the importance of understanding the complexities around adolescent involvement in care and decision-making with the goal of improving adolescent health outcomes in pediatric advanced cancer.

## Figures and Tables

**Table 1.** Participant characteristics (*N* = 41)

Variable	*N* (%)	*M* (*SD*)
Age (years)	-	15.37 (3.34)
Time since initial diagnosis (months)	-	37.83 (49.15)
Sex		
Male	27 (65.9)	-
Female	14 (34.1)	-
Race		
Asian	3 (7.3)	-
Black or African American	1 (2.4)	-
White	33 (80.5)	-
Multi-race or not listed	4 (9.8)	-
Ethnicity		
Not Hispanic or Latino	41 (100)	-
Diagnosis type		
Leukemia	14 (34.1)	-
Lymphoma	3 (7.3)	-
Brain tumor	4 (9.8)	-
Other solid tumor	20 (48.8)	-
Family income		
Under $25,000 per year	11 (28.9)	-
$25,001-$50,000 per year	6 (15.7)	-
$50,001-$75,000 per year	5 (13.2)	-
$75,001-$100,000 per year	6 (15.7)	-
$100,001-$150,000 per year	6 (15.7)	-
Over $150,000 per year	4 (10.5)	-

**Table 2.** Codebook used for qualitative analysis of adolescent report of family treatment decision-making and factors considered

Code	Definition	Purpose/meaning of code	Examples
*Decision-making codes*			
1) Active decision involvement	The adolescent describes themselves as taking an active role in decision-making, having a significant influence in decisions.	*Does the adolescent have a high level of involvement in treatment decision-making?*	“I talk to my parents about it and they tell me what they think and then I make a decision.”
2) Collaborative decision involvement	The adolescent describes the decision-making process as a collaboration between themselves, their caregivers, medical team, and/or other family members.	*Does the adolescent have meaningful involvement or consultation in treatment decision-making?*	“The doctor tells us what our options are and then my parents talk about it with me and ask what I think and then they make a decision.”
3) Passive decision involvement	The adolescent describes themselves as having little to no involvement, either by choice or default.	*Is the adolescent removed from the treatment decision-making process?*	“My parents listen to the doctor and decide what to do.” or “I just let my parents decide.”
*Intrinsic decision factors*			
4) Side effects	The adolescent references side effects as an influential decision-making factor	*Are symptoms or treatment side effects influencing the treatment decision-making process?*	“Side effects or how the treatment will make me feel.” “How treatment will affect me like long-term and stuff.”
5) Chance of cure	The adolescent references the likelihood of treatment curing their disease as an influential decision-making factor	*Is the treatment effectiveness influencing the treatment decision-making process?*	“Which one works the best.” “If it’s going to work.”
6) Length of treatment	The adolescent references the length of treatment or time that would have to be spent at the hospital as an influential decision-making factor	*Do adolescents consider the length of treatment or time spent at the hospital when making treatment decisions?*	“How long I'll have to be in the hospital.” “I have to make sure it doesn’t get in the way of other appointments or things my parents have to do.”
7) Don’t know/Wasn’t asked	The adolescent says they don’t know what factors are considered when making decisions or they were not asked.	Including cases where adolescent may not be involved in decisions or was not asked about the factors they consider.	
*Extrinsic decision-factors*			
8) Family impact	The adolescent references the stress or financial burden that treatment may place on their family as a decision-making factor.	*Are the potential impacts their family influencing factors for adolescent treatment decision-making?*	“If it would cost a lot of money.” “How worried my parents would be.”
9) Family thoughts	The adolescent says the opinions of their parents, siblings, or other extended family members are a factor in their decision-making.	*Do the opinions of family members factor into adolescent treatment decision-making?*	“What my parents think I should do.”
10) Other outside influencers	The adolescent references factors outside of their family, such as cultural beliefs, their health-care team's opinion, or their friends, as factors influencing their decision-making.	*Are there factors outside of the family that adolescents consider when making treatment decisions?*	“What the doctors say we should do.” “My religion says I can’t do certain treatments.”

**Table 3.** Hierarchical linear regression predicting adolescents’ frequency of and desired change in decision involvement

Variables	Frequency of involvement			Desire for change in involvement		

	*B* (SE)	*β*	*p*	*B* (SE)	*β*	*p*

*Step 1*						

Constant	−.041 (.524)	.486	.938	.727 (.272)	.208	.011
Adolescent age	.114 (.033)		.001***	.022 (.017)		.199
	*F* = 11.773***			*F* = 1.710		
	*R*^2^ = .237			*R*^2^ = .043		

Step 2						

Constant	−.135	.462	.819	.559 (.31)	.114	.067
Adolescent age	.108	.060	.005**	.012 (.02)	.232	.511
Autonomy attitudes	.063		.704	.113 (.08)		.184
	*F* = 5.828**			*F* = 1.792		
	*R*^2^ = .240			*R*^2^ = .088		

*Step 3*						

Constant	.406 (.634)	.539	.527	.556 (.308)	.260	.075
Adolescent age	.126 (.039)	.066	.003**	.028 (.02)	.361	.142
Autonomy attitudes	.069 (.167)	−.228	.683	.176 (.081)	.206	.038*
Extrinsic decision factors	−.157 (.098)	−.140	.119	.066 (.048)	−.439	.178
Intrinsic decision factors	−.127 (.151)		.406	−.184 (.073)		.017*
	*F* = 3.995**			*F* = 2.858*		
	*R*^2^ = .313			*R*^2^ = .246		

*Step 4*						

Constant	.131 (1.628)	.539	.937	.523 (.791)	.260	.148
Adolescent age	.126 (.039)	.078	.003**	.028 (.019)	.365	.055
Autonomy attitudes	.082 (.184)	−.232	.658	.178 (.089)	.205	.190
Extrinsic decision factors	−.159 (.101)	−.147	.122	.065 (.049)	−.441	.020*
Intrinsic decision factors	−.133 (.157)	.029	.401	−.185 (.076)	.010	.953
Agreement with parents on	.055 (.301)		.855	.009 (.146)		
goals of care	*F* = 3.115*			*F* = 2.222		
	*R*^2^ = .314			*R*^2^ = .246		

* *p* < .05,

** *p* < .01,

*** *p* < .001.

**Table 4.** integrated display of adolescent decision involvement

Domain	Qualitative investigation	Frequency (%)	Quantitative investigation	Mixed-method interpretation
Active decision involvement	“The treatment decision was my decision along with my fiancé, that’s what my parents wanted, um, they-they recommended which treatment they saw best but ultimately they were like hands off they were like you make the final decision.” (23-year-old female, Ewing's sarcoma) “I just said, I feel like I should do this. Like ... I don’t want this to affect my college career plans and stuff. So my dad was like. I'm kind of worried you're doing it, doing it during covid. But if you really want to do it, I really don’t have much say in it.” (18-year-old female, relapsed chronic myeloid leukemia)	17	Mean age: 19.4 Mean CIQ-IF: 3.67 Mean FRQ-frequency: 2.67	Quantitatively, value of intrinsic factors relates to a higher level of adolescent involvement in decision-making. However, qualitative responses suggest that value of intrinsic factors only relates to passive vs. non-passive decision involvement. Qualitative findings do align with quantitative reports of decision involvement.
Collaborative decision involvement	“Um, me and my mom make the decisions like we get, um, opinions from our family so like we'll sit down and kind of have like a family meeting and talk about it and just to inform them. But me and my mom are pretty much the ones who make the decisions ... Like sometimes I want things that my mom's like 'wait, you need to like wait it out and think about it.' ... Like with the chemo I was supposed to do another round of chemo not too long ago but I held off on it because I wanted to” (17-year-old female, mixed germ cell tumor) “Like if it involves me, my mom and dad just ask me what I think and what I would do ... So they don’t really make a decision without telling me and asking me what I want to do.” (16-year-old male, acute myeloid leukemia)	53	Mean age: 15.5 Mean CIQ-IF: 3.49 Mean FRQ-frequency: 1.69	
Passive decision involvement	“Most of it’s my mom, but I mean, she'll run things by my dad to just to make sure that, you know, they don’t mess with his schedule. I mean, I'd like to say, I feel like I have a say in it, but most of it’s handled, you know, through my mom.” (15-year-old male, anaplastic ganglioglioma) “I ask my dad and mom what I- what my decisions are. Um, they help me figure out, like, what I should do, not to do.” (11-year-old male, anaplastic large cell lymphoma)	30	Mean age: 12.33 Mean CIQ-IF: 2.77 Mean FRQ-frequency: 1.37	

## Abbreviations.

QOL: Quality of life
EOL: end-of-life
C-CIQ: Cancer Information Questionnaire for Adolescents
AISPS: Autonomy and Information-Seeking Preference Scale
FRQ: Family Roles Questionnaire
